# Correction: García-Aliaga et al. Comparative Analysis of Soccer Performance Intensity of the Pre–Post-Lockdown COVID-19 in LaLiga™. *Int. J. Environ. Res. Public Health* 2021, *18*, 3685

**DOI:** 10.3390/ijerph19020999

**Published:** 2022-01-17

**Authors:** Abraham García-Aliaga, Moisés Marquina, Antonio Cordón-Carmona, Manuel Sillero-Quintana, Alfonso de la Rubia, Silvestre Jos Vielcazat, Fabio Nevado Garrosa, Ignacio Refoyo Román

**Affiliations:** 1 Facultad de Ciencias de la Actividad Física y del Deporte (INEF-Sports Department), Universidad Politécnica de Madrid, 28040 Madrid, Spain; abraham.garciaa@upm.es (A.G.-A.); antoupm@gmail.com (A.C.-C.); manuel.sillero@upm.es (M.S.-Q.); alfonso.delarubia@upm.es (A.d.l.R.); ignacio.refoyo@upm.es (I.R.R.); 2 Department of Competitions and Mediacoach, LaLiga, 28043 Madrid, Spain; sjos@laliga.es (S.J.V.); fnevado@laliga.es (F.N.G.)

The authors wish to make the following corrections to this paper [1]: 

## Addition of Authors

Silvestre Jos Vielcazat and Fabio Nevado Garrosa were not included as co-authors in the published article. The corrected author contributions statement appears below.

Conceptualization, A.G.-A., M.M. and I.R.R.; methodology, A.G.-A., A.C.-C. and M.S.-Q.; software, A.G.-A. and A.C.-C.; validation, M.M., M.S.-Q. and I.R.R.; formal analysis, A.G.-A., M.M. and A.C.-C.; investigation, A.G.-A., M.M. and I.R.R.; resources, A.G.-A., S.J.V., F.N.G. and A.d.l.R.; data curation, M.M., A.C.-C., S.J.V., F.N.G. and M.S.-Q.; writing—original draft preparation, A.G.-A., M.M. and A.C.-C.; writing—review and editing, M.S.-Q., A.d.l.R. and I.R.R.; visualization, A.G.-A.; supervision, I.R.R.; project administration, I.R.R.; funding acquisition, I.R.R. All authors have read and agreed to the published version of the manuscript.

## Text Corrections

The first and second paragraph of Section 2.2. should be: Data were obtained from LaLiga, which authorised the use of the variables included in this investigation. Data were extracted by a valid and reliable multicamera tracking system and associated software (Mediacoach^®^, Madrid, Spain). Mediacoach^®^ records the position of each player on the pitch at 25 Hz using a stereo multi-camera system composed of two multi-camera units placed on either side of the midfield line. Each multi-camera unit contains three cameras with a resolution of 1920 × 1080 pixels, which are synchronised to provide a stitched panoramic picture [24]. The analysis included physical performance data that were evaluated by a computerised multi-camera tracking system (TRACAB^®^, Stockholm, Sweden) that had a sampling frequency of 25 Hz. This tracking system semi-automatically evaluated the match performance data of all players, the position of the ball, and the corresponding match events, allowing the intensity of the run to be quantified. The validity of Mediacoach^®^ to assess running distance during soccer match play has been obtained through a high agreement with data obtained using global positioning system units [25,26] and data obtained from a reference camera system (i.e., VICON motion capture system [27]).

The sixth paragraph of the Discussion section should be: The similarities in the length of the interruption of the competition due to COVID-19 in Spain and the summer vacations between soccer seasons indicate that the players face a similar scenario at the beginning of the soccer season. This was similar in terms of time, but not in the case of training, in which the confinement situation was quite far from standard. There were also differences during the ‘pre-season’ due to the protocols that had to be followed in the different LaLiga^TM^ teams. It has been hypothesised that when the competition was resumed after the COVID-19 lockdown, professional LaLiga^TM^ players experienced physical challenges similar to those they usually experience during the first official matches of the season. The results of this study differ from those found by Brito Souza, López-Del Campo, Blanco-Pita, Resta, and Del Coso [32], which showed that, in the previous four competitive seasons of LaLiga^TM^, the physical performance of the players was lower at the beginning of the season, and the teams needed approximately 8–10 match days to achieve constant performance.

The eighth paragraph of the Discussion section should be: Teams are already established in terms of tactics and strategies, but if not trained, team coordination can be affected. An important aspect was that the training protocols established by health authorities for all were the same in terms of the training situations that could occur. In addition, the players—first individually, then as a group, and then collectively—were preparing for the competition. The level of training volume differed from other pre-seasons. However, the types of exercises were not related to one another when approaching or moving away from the specifics of the game. This influences not only physical fatigue but also psychological fatigue, generating much more stress and a feeling of intensity if training is done in isolation. A very important factor was that these collective situations could not be addressed in friendly matches, something that is done in the pre-season. Therefore, the physiological and tactical volume was affected. In this way, the type of training influences the demands encountered in competition. The return to training protocol designed by LaLiga under the recommendations/limitations of the health authorities affects the training and, therefore, the conditional performance during the competition. In this context of limited training sessions, in terms of the number of participants and the limited number of preparation matches, there has been an increase in the number of small-space tasks with few players and a decrease in the number of long-distance tasks. In this sense, tasks with few players and a relatively small space per player could be an indicator of a deficit in the distances covered at high speeds; it is logically hard to reach high speeds in a small space [34,35]. Moreover, training with small groups of players, usually through tasks with more space limitations, promotes better adaptation to the ability to accelerate and decelerate repeatedly, which could be another reason for an increase in accelerations and decelerations per minute [36–38].

The first sentence of the Conclusions section should be: Indicators of physical performance in LaLiga^TM^ matches after confinement due to COVID-19 varied the conditional response in competition because of limitations in training methods during the ‘pre-season’ without experiencing low or negative performance in competition.

## Addition of References

New references [24–27,34–38] should be added.

24 Del Coso, J.; Brito de Souza, D.; Moreno-Perez, V.; Buldú, J.M.; Nevado, F.; Resta, R.; López-Del Campo, R. Influence of players’ maximum running speed on the team’s ranking position at the end of the Spanish LaLiga. *Int. J. Environ. Res. Public Health* **2020**, *17*, 8815, https://doi.org/10.3390/ijerph17238815.
25 Pons, E.; García-Calvo, T.; Resta, R.; Blanco, H.; López del Campo, R.; Díaz García, J.; Pulido, J.J. A comparison of a GPS device and a multi-camera video technology during official soccer matches: Agreement between systems. *PLoS ONE* **2019**, *14*, e0220729, https://doi.org/10.1371/journal.pone.0220729.
26 Felipe, J.L.; Garcia-Unanue, J.; Viejo-Romero, D.; Navandar, A.; Sánchez-Sánchez, J. Validation of a video-based performance analysis system (Mediacoach^®^) to analyze the physical demands during matches in LaLiga. *Sensors* **2019**, *19*, 4113, https://doi.org/10.3390/s19194113.
27 Linke, D.; Link, D.; Lames, M. Football-specific validity of TRACAB’s optical video tracking systems. *PLoS ONE* **2020**, *15*, e0230179, https://doi.org/10.1371/journal.pone.0230179.
34 Dellal, A.; Varliette, C.; Owen, A.; Chirico, E.N.; Pialoux, V. Small-sided games versus interval training in amateur soccer players. *J. Strength Cond. Res.* **2012**, *26*, 2712–2720, https://doi.org/10.1519/JSC.0b013e31824294c4.
35 Nevado-Garrosa, F.; Torreblanca-Martínez, V.; Paredes-Hernández, V.; del Campo-Vecino, J.; Balsalobre-Fernández, C. Effects of an eccentric overload and small-side games training in match accelerations and decelerations performance in female under-23 soccer players. *J. Sports Med. Phys. Fit.* **2021**, *61*, 365–371, https://doi.org/10.23736/S0022-4707.20.11232-5.
36 Hodgson, C.; Akenhead, R.; Thomas, K. Time-motion analysis of acceleration demands of 4v4 small-sided soccer games played on different pitch sizes. *Hum. Mov. Sci.* **2014**, *33*, 25–32, https://doi.org/10.1016/j.humov.2013.12.002.
37 Gaudino, P.; Alberti, G.; Iaia, F.M. Estimated metabolic and mechanical demands during different small-sided games in elite soccer players. *Hum. Mov. Sci.* **2014**, *36*, 123–133, https://doi.org/10.1016/j.humov.2014.05.006.
38 Rebelo, A.N.C.; Silva, P.; Rago, V.; Barreira, D.; Krustrup, P. Differences in strength and speed demands between 4v4 and 8v8 small-sided football games. *J. Sports Sci.* **2016**, *34*, 2246–2254, https://doi.org/10.1080/02640414.2016.1194527.

The authors apologize for any inconvenience caused and state that the scientific conclusions are unaffected. The original article has been updated.
